# One-year outcomes of a surgical technique for stabilizing the intraocular lens–capsular bag complex in severely subluxated cataracts using permanent polypropylene capsular hooks

**DOI:** 10.3389/fmed.2025.1574908

**Published:** 2025-09-24

**Authors:** Rui Feng, Guoliang Li, Yanni Luo, Binjian Wang, Yihao Zhao, Chao Qu

**Affiliations:** ^1^Department of Ophthalmology, Sichuan Provincial People’s Hospital, University of Electronic Science and Technology of China, Chengdu, China; ^2^Eye School of Chengdu University of Traditional Chinese Medicine, Chengdu, China

**Keywords:** severely subluxated cataracts, IOL–capsular bag complex, permanent polypropylene capsular hook, case series, surgical technique

## Abstract

**Purpose:**

To report the one-year follow-up outcomes of a surgical technique designed to rescue severely subluxated cataracts and stabilize the intraocular lens (IOL)–capsular bag complex using permanent polypropylene capsular hooks.

**Methods:**

Four patients (four eyes) with severely subluxated cataracts underwent surgery. A spatula was used to elevate and stabilize the dislocated lens in the retropupillary space to facilitate capsulorhexis. Phacoemulsification and aspiration were performed using capsular retractors and a capsular tension ring. Permanent polypropylene capsular hooks were implanted to stabilize the IOL–capsular bag complex. Postoperative outcomes were assessed over a follow-up period of at least 1 year. Descriptive analyses were performed to summarize and report the outcomes of this limited case series.

**Results:**

The follow-up period ranged from 13 to 20 months. All patients demonstrated improved and stable subjective refraction and best-corrected visual acuity (BCVA). At the final follow-up, the mean postoperative prediction error was −0.17 ± 0.29 D. Slit-lamp examinations showed that the IOL–capsular bag complex and the permanent polypropylene capsular hooks remained stable. Anterior segment optical coherence tomography (AS-OCT) revealed minimal IOL tilt of 1.52 ± 1.20. No intraoperative or postoperative complications were reported.

**Conclusion:**

The technique offers a viable approach for managing severely subluxated cataracts, providing stable support for the IOL–capsular bag complex and yielding favorable clinical outcomes. However, as this novel technique was evaluated in a small sample size, further studies with larger cohorts and longer follow-up periods are needed to confirm its long-term safety and efficacy.

## Introduction

The surgical management of severely subluxated cataracts remains challenging, even for experienced anterior segment surgeons. These cases are associated with a high risk of complications such as capsular rupture or displacement of lens material into the vitreous cavity (1, 2).

Current surgical strategies for managing severely subluxated cataracts include (3) either removal of the entire lens and implantation of an intraocular lens (IOL) using various fixation techniques (e.g., anterior chamber IOL, transscleral fixation, intrascleral fixation, or iris fixation) (4–7), or preservation of the capsular bag with the use of endocapsular supporting devices (e.g., capsular tension ring [CTR], glued capsular hook, or capsular anchor), followed by in-the-bag IOL implantation (8–11).

Preserving an intact capsule bag helps maintain anterior and posterior segment anatomy, thereby reducing the risks of glaucoma and retinal complications (3, 12). Moreover, capsular bag preservation allows for in-the-bag IOL placement, which minimizes uveal chafing and reduces the risk of iris capture (12–14). Current consensus recommends preserving the capsular bag and performing intracapsular IOL implantation whenever feasible. However, maintaining capsular bag integrity in severely subluxated cataracts remains technically difficult.

Levitation of the lens into the retropupillary space is critical to achieving a successful capsulorhexis and preserving the capsular bag. Several levitation techniques have been introduced. Injection of viscoat or perfluorocarbon liquids into the vitreous cavity has been reported, but these approaches carry risks such as intraocular pressure (IOP) elevation and potential ocular toxicity (15, 16). The phacotip-assisted technique is more appropriate for managing dropped nuclei (17). In contrast, posterior-assisted levitation does not require intraocular injection into the vitreous cavity, allowing phacoemulsification while maintaining anatomical structures. For patients whose nucleus is located in the posterior chamber behind the pupil and are suitable for in-the-bag IOL implantation, posterior-assisted levitation offers a simple and accessible technique (18).

After lens levitation, the use of capsular supporting devices is essential to stabilize the IOL. Compared with other support techniques (such as glued capsular hooks, modified CTRs, or capsular tension segments), permanent polypropylene capsular hooks eliminate the need for scleral flaps or complex suturing, are easy to maneuver and reduce operative time. Thus, in this study, we report the one-year outcomes of a surgical technique that combines posterior-assisted levitation with permanent polypropylene capsular hook implantation for the management of severely subluxated cataracts. This technique aims to preserve anatomical structures and restore visual function, providing anterior segment surgeons with a simple and effective surgical option for managing the severely subluxated cataracts.

## Methods

### Patients

This study was conducted at Sichuan Provincial People’s Hospital (Chengdu, China) and approved by the Ethics Committee of Sichuan Provincial People’s Hospital. Informed consent was obtained from all patients. This case series was reported in accordance with the CARE (CAse REport) guidelines.

Patients diagnosed with severely subluxated cataracts (zonular dialysis ranging from 6 to 9 clock hours) were included in this study. The extent of zonular dialysis was assessed by the surgeon based on the lens displacement observed through a dilated pupil in the supine position. Eligible patients were required to have a clearly visible lens in the posterior chamber, directly behind the pupil. Patients were excluded if they had other ocular diseases, a history of prior ocular surgery, or any systemic disease.

All patients underwent comprehensive ophthalmic evaluations, including best-corrected visual acuity (BCVA), subjective refraction, IOP, slit-lamp examination, dilated fundus examination, Pentacam (Oculus, Wetzlar, Germany) and B-scan ultrasonography (Aviso, Quantel Medical, France). Axial length (AL), anterior chamber depth (ACD), lens thickness (LT), white-to-white corneal diameter, and keratometry values (flat keratometry and steep keratometry, K1 and K2) were measured using the IOL Master 700 (Carl Zeiss Meditec, Jena, Germany). Endothelial cell density (ECD) was measured using the CEM-530 (Nidek, Aichi, Japan).

IOL tilt was measured using anterior segment optical coherence tomography (AS-OCT) with the VG200D (SVision Imaging, Henan, China). Radial scans were performed following pharmacologic pupil dilation. A vertical scan obtained from AS-OCT was selected for analysis. A reference line was drawn between the iridocorneal angles. The angle between the reference line and the horizontal axis of the IOL was defined as the IOL tilt. Based on existing model eye and clinical studies, an IOL tilt of ≥7 degrees was considered clinically significant (19–21).

### Surgical technique

All surgeries were conducted under retrobulbar anesthesia. A microvitreoretinal blade was used to create a perpendicular scleral incision, approximately 3.5 mm posterior to the corneal limbus. A spatula was inserted through this incision to gently elevate the subluxated lens into the retropupillary space. Capsulorhexis was performed through the corneal incision on the elevated lens. Capsule retractors were inserted via corneal incisions and positioned at the capsular margin. A CTR was implanted into the capsular bag. Hydrodissection, phacoemulsification and cortical aspiration were performed using the retractors and CTR.

**FIGURE 1:**
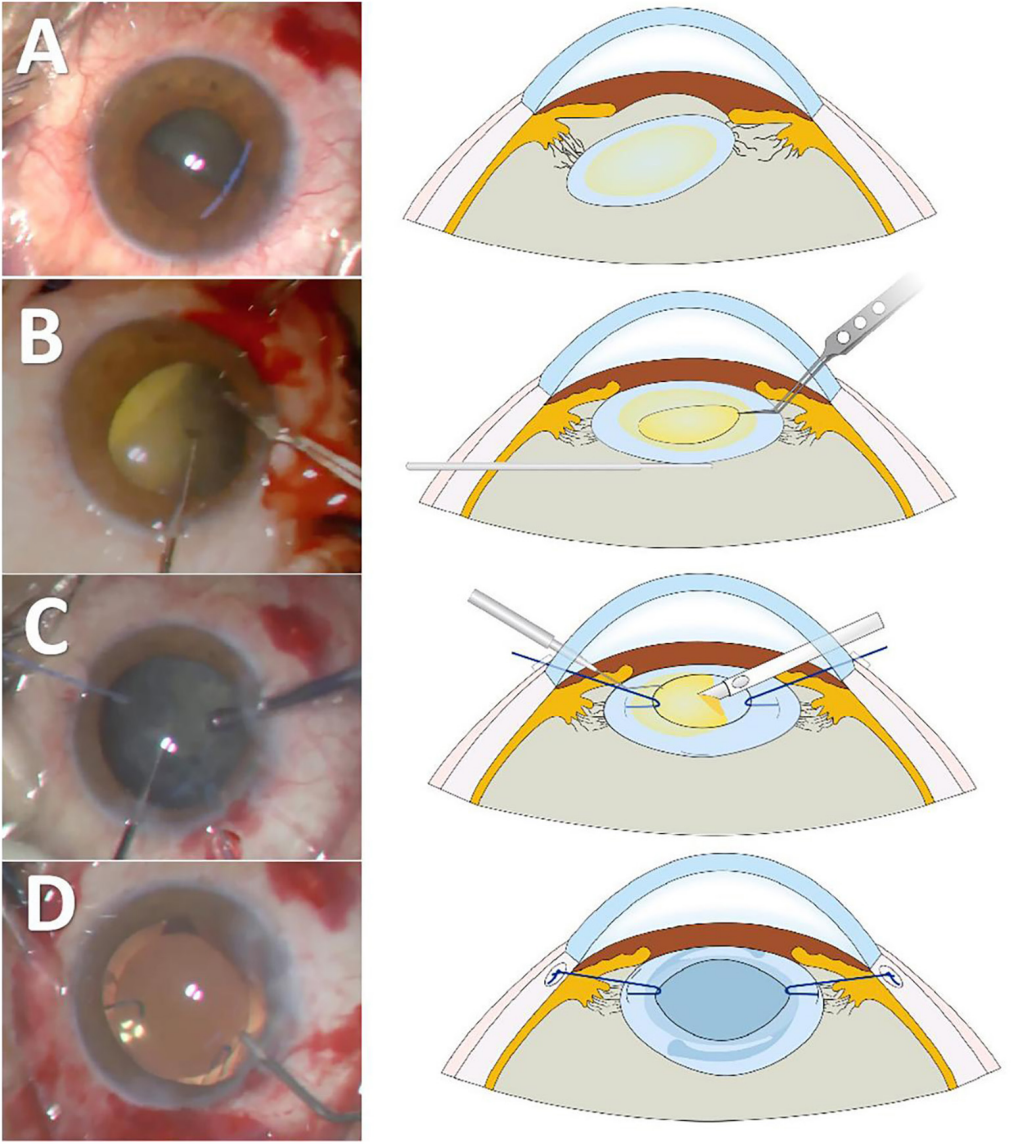
Main surgical steps and schematic illustration of the technique. **(A)** Severely subluxated cataracts with minimal zonular support. **(B)** A spatula is used to elevate and stabilize the lens into the retropupillary space to facilitate capsulorhexis. **(C)** A capsule retractor suspends and stabilizes the capsular bag, enabling safe phacoemulsification and aspiration. **(D)** Permanent polypropylene capsular hooks are used to stabilize the intraocular lens (IOL)–capsular bag complex.

Permanent polypropylene capsular hooks were fashioned from 6–0 non-absorbable polypropylene suture material with a curved needle. A thermoplastic bend was created by heating the suture at the turning point. The distal end was trimmed approximately 2.0 mm from the bend to form the hook (Supplementary Video 1). The hook was inserted via a corneal incision, docked into a 27-gauge needle introduced through the ciliary sulcus posterior to the limbus and externalized through the sclera. The externalized tip was secured using the Z-suture technique. Gentle tension was applied during trimming to facilitate retraction of the suture end into the sclera, ensuring that it remained buried. The IOL was inserted into the preserved capsular bag and centered after placement of the permanent polypropylene capsular hook. Following viscoelastic removal, carbachol was administered for myosis. Postoperatively, topical antibiotics and corticosteroids were prescribed (Figure 1 and Supplementary Video 2).

### Data analysis

Given the small sample size and case series design, all analyses were descriptive and no formal hypothesis testing was performed. Continuous variables were reported as mean ± standard deviation (SD). Categorical variables were summarized by frequencies.

## Results

Four patients (four eyes) with severely subluxated cataracts, including two females and two males, were included (Table 1). The average patient age was 66.00 ± 8.68 years. All cases involved blunt ocular trauma and presented with decreased visual acuity. The mean preoperative BCVA was 0.51 ± 0.35 LogMAR. Zonular dialysis ranged from 6 clock hours in Case 2 (from 10 o’clock to 4 o’clock), to 7 clock hours in Case 1 (from 10 o’clock to 5 o’clock) and Case 4 (from 12 o’clock to 7 o’clock), and 9 clock hours in Case 3 (from 10 o’clock to 7 o’clock).

**TABLE 1:** Patient demographic and clinical data.

Case	1	2	3	4	Mean ± SD
Sex	Female	Male	Male	Female	NA
Eye	Left	Left	Right	Left	NA
Age (years)	77	67	64	56	66.00 ± 8.68
Etiology	Injured by a wire	Injured by a massage device	Injured by a nail	Injured by hand	NA
Nuclear density^a^	4	3	3	3	3.25 ± 0.50
Intraocular pressure (mmHg)	12	15	20	17	16.00 ± 3.37
Follow-up time (months)	13	14	20	15	15.50 ± 3.11

SD, standard deviation; NA, not applicable.

^a^Cataract grade according to Lens Opacities Classification System III, nuclear score. All data analyses were descriptive and no formal hypothesis testing was performed.

All patients underwent the posterior-assisted levitation followed by the implantation of permanent polypropylene capsular hooks. In three cases with 6–7 clock hours of zonular dialysis, one permanent polypropylene capsular hook was used. In the case with 9 clock hours of zonular dialysis, two permanent polypropylene capsular hooks were used. No intraoperative complications were encountered.

The mean follow-up time was 15.50 ± 3.11 months. All patients exhibited improvement in both subjective refraction and BCVA during the one-year follow-up. The mean postoperative prediction error was −0.17 ± 0.29D, indicating satisfactory refractive accuracy for the four patients (Table 2).

**TABLE 2:** Follow-up data of four patients.

Case		1	2	3	4	Mean ± SD
BCVA (LogMAR)	Pre	0.52	1.00	0.22	0.30	0.51 ± 0.35
	Post	0.10	0	0	0	0.03 ± 0.05
Subjective refraction (D)	Pre	−0.00DS 1.25DC × 120	NA	−0.50DS	−0.50DS −1.50DC × 65	NA
	Post	−0.00DS −0.50DC × 95	+ 1.25DS −2.00DC × 95	−0.25DS −0.75DC × 120	−0.00DS −0.50DC × 75	NA
Corneal endothelial density (Cells/mm^2^)	Pre	2801	2777	2748	2628	2738.50 ± 76.79
	Post	2562	2534	2423	2431	2487.50 ± 70.86
Prediction error (D)		−0.30	0.25	−0.395	−0.23	−0.17 ± 0.29
IOL tilt (degree)		0.86	3.20	0.50	1.50	1.52 ± 1.20

SD, standard deviation; D, diopter; NA, not applicable; BCVA, best-corrected visual acuity; IOL, intraocular lens; DS, diopters sphere; DC, diopters cylinder. All data analyses were descriptive and no formal hypothesis testing was performed.

**FIGURE 2:**
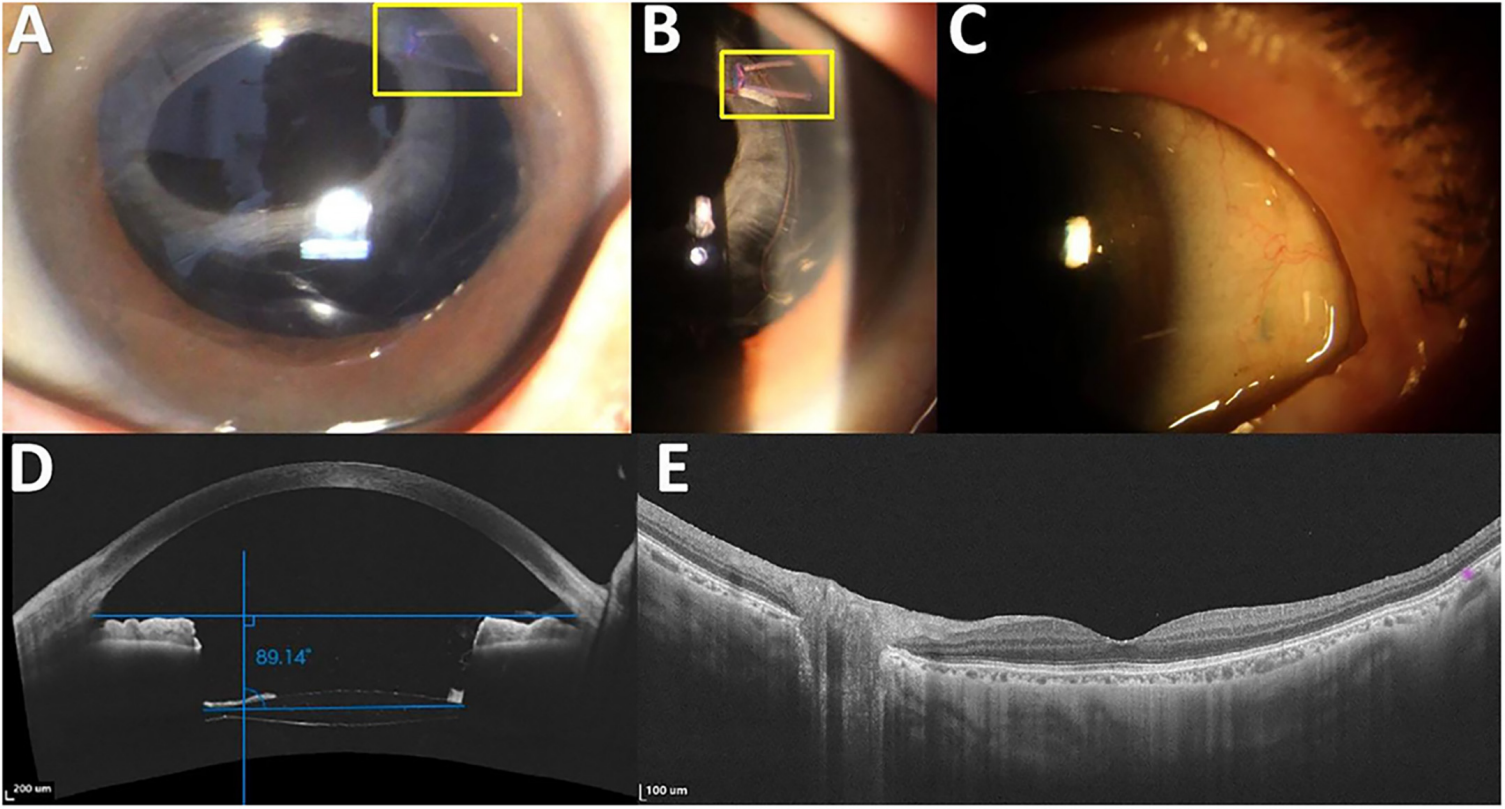
Surgical outcome in Case 1. **(A)** The intraocular lens (IOL)–capsular bag complex remains well-centered within the pupil, with the permanent polypropylene capsular hooks *in situ*. **(B)** Permanent polypropylene capsular hooks. **(C)** No suture exposure, erosion, or conjunctival inflammation is observed. **(D)** Anterior-segment optical coherence tomography (AS-OCT) image showing a well-centered IOL–capsular bag complex. The top horizontal blue line represents the reference line between the iridocorneal angles, whereas the bottom horizontal blue line represents the horizontal axis of the IOL. The angle between these two lines, relative to 90 degrees, indicates the IOL tilt (89.14 degrees in this example, minus 90 degrees, corresponding to a tilt of 0.86 degrees). **(E)** Macular OCT image showing no significant abnormalities during a follow-up period of at least 1 year. The yellow box highlights the permanent polypropylene capsular hook.

On slit-lamp examination, all IOLs remained well-centered and the permanent polypropylene capsular hooks remained *in situ*. AS-OCT confirmed a mean IOL tilt of 1.52 ± 1.20, supporting stable postoperative IOL positioning over the one-year follow-up for the four patients. Mean postoperative corneal endothelial cell density loss was 9.15 ± 1.87%. No cases of suture exposure, conjunctival inflammation, or fundus abnormalities were observed during follow-up (Figure 2).

## Discussion

The primary goals in managing severely subluxated cataracts are safe lens removal and stable IOL fixation. In this study, we report a combined technique incorporating posterior-assisted levitation, phacoemulsification with capsular retractors and a CTR, and in-the-bag IOL implantation. This approach offers a conservative and effective solution for achieving these surgical objectives.

Posterior-assisted levitation facilitates a capsular-sparing phacoemulsification, which preserves anatomical structures and enables in-the-bag IOL implantation. The capsular-sparing phacoemulsification minimizes the risk of nuclear fragment dislocation into the vitreous and reduces the need for pars plana vitrectomy. Additionally, preserving the integrity of the capsular bag facilitates in-the-bag IOL implantation. In-the-bag IOL implantation reduces the likelihood of complications associated with alternative fixation techniques such as IOL optic capture or pseudophakic bullous keratopathy. Previous studies have demonstrated that posterior-assisted levitation could achieve safe and encouraging clinical outcomes (22, 23). Consistent with these findings, our patients showed favorable outcomes without significant complications over the one-year follow-up. However, extended follow-up with larger sample sizes is needed to evaluate the long-term safety and effectiveness.

Postoperative IOL stability is critical to achieving accurate and stable refractive outcomes in patients with severely subluxated cataracts. Previously described supporting techniques, including glued capsular hooks, modified CTRs, or capsular tension segments (3, 8), often involve scleral manipulations (scleral flaps or tunnels) or complicated knotting techniques. In contrast, permanent polypropylene capsular hooks eliminate the need for scleral flaps or complex suturing, reducing both surgical time and complexity. In our study, all patients had a postoperative refractive prediction error within ± 0.5D, suggesting high prediction accuracy. In addition, AS-OCT demonstrated minimal IOL tilt, indicating stable IOL positioning. This may result from the synergistic effect of the CTR and the permanent polypropylene capsular hooks. The CTR counters the capsular bag contraction and maintains IOL centration. The permanent polypropylene capsular hooks provide external support to compromised zonular regions, thereby stabilizing the IOL–capsular bag complex. These findings suggest that the permanent polypropylene capsular hooks, which are accessible and cost-effective, offer a valuable option for anterior segment surgeons. Nevertheless, potential suture-related complications, including suture degradation, erosion, or late-onset instability of the IOL–capsular bag complex, warrant long-term follow-up.

Several key steps should be considered to improve the surgical success with this technique. A comprehensive preoperative assessment of lens subluxation in both sitting and supine positions is crucial. This method is not suitable for patients with progressive lens subluxation, such as those with Marfan syndrome. Additionally, a well-centered and properly sized continuous curvilinear capsulorhexis is essential to facilitate effective placement of polypropylene capsular hooks and ensure IOL–capsular bag complex stability. In our cases, the Z-suture technique was used to secure the hooks and minimize suture-related complications (24, 25). Creating a flange at the suture end, similar to the Yamane technique, may further reduce the risk of suture exposure. These technical refinements would contribute to the absence of postoperative complications in our study.

This study has several limitations. As a small case series, it describes a simple and accessible technique for managing severely subluxated cataracts. Although the one-year follow-up demonstrated favorable safety and stability, further studies with larger sample sizes and longer durations are warranted to determine the broader applicability and long-term outcomes. Moreover, this descriptive report lacks comparisons with other conventional surgical techniques. Future controlled studies are required to evaluate the comparative benefits and efficacy of this method. Additionally, inclusion was limited to cases with 6–9 clock hours of zonular dialysis, potentially introducing selection bias.

Surgical management of severely subluxated cataracts remains challenging, especially in resource-limited developing countries. In many cases, vitreoretinal surgery is often performed to restore visual function (26). Our novel technique enables eligible patients to undergo anterior segment surgery with preservation of the capsular bag, thereby reducing both the economic and psychological burdens on patients. This approach facilitates anterior segment reconstruction with satisfactory visual outcomes in patients with severely subluxated cataracts. One-year follow-up suggests good safety and stability. However, further studies with longer follow-up periods and larger sample sizes are necessary to establish the generalizability of this technique.

## Data Availability

The raw data supporting the conclusions of this article will be made available by the authors, without undue reservation.

## Supplementary material

Supplementary Video 1 Demonstration of the procedure for creating a permanent polypropylene capsular hook.

Supplementary Video 2 Surgical technique demonstrating the rescue of a severely subluxated cataract using permanent polypropylene capsular hooks to stabilize the intraocular lens (IOL)–capsular bag complex.
